# Supplementary material for: Machine learning-based predictions of dietary restriction associations across ageing-related genes
Source: BMC Bioinformatics. 2022 Jan 4;23:10. doi: 10.1186/s12859-021-04523-8 (PMC8729156; doi:10.1186/s12859-021-04523-8)
Supplement: Supplementary file 1 — Additional file 1: Text S.1.1. KEGG-Pertinence Dataset construction. Text S.1.2. Protein descriptors. Text S.2. Hyperparameters Grid. Text S.3. Analysis of GenDR (Gene expression) genes based on DR-association probability. Text S.4. KEGG-Pertinence. Text S.5. KEGG-Influence. Table S1. Classification of amino acids into three possible functional groups according to their corresponding physicochemical properties. Table S2. Ensemble algorithms’ hyperparameters grid. Ensemble algorithms’ hyperparameters grid for sampling strategy, maximal number of features allowed in each base estimator, number of base estimators, class weight and binary threshold. The definition of αus is as expressed in equation (S.1). The number n in Max features is the same as the total number of features in the dataset of interest. The last column shows the algorithm(s) that use each of the hyperparameters mentioned in the first column. Table S3. DR-probability analysis of the subset of ageing-related genes that are differentially expressed during DR. Table S4. Top-seven most predictive pathways by the {KEGG-Pertinence, BRF} model. Table S5. Top-ten most predictive nodes by the {KEGG-Influence, XGB} model. Figure S1. ROC curve and AUC of the ML algorithms on the two most predictive datasets. Figure S2. Comparison of the two most predictive models. A and B, confusion matrices of {GO terms, BRF} and {PathDIP, CAT}, respectively. Figure S3. Overlapping between ageing-related, -related and -related genes. [file 12859_2021_4523_MOESM1_ESM.pdf]

RESEARCH

Supplementary material: Machine learning-based predictions of dietary restriction associations across ageing-related genes

Supplementary texts

S.1 More on Datasets Constructions

S.1.1 KEGG-Pertinence Dataset construction

KEGG pathways can be regarded as directed graphs where each node N is typically associated with either one gene product (*e.g.*, $N = \{G_1\}$) or more (*e.g.*, $N = \{G_1, G_2, G_3, \dots G_n\}$), provided that the set of genes contained in a single node share similar functions and interactions. By contrast, edges represent how different nodes interact, mainly mediated by post-translational modifications and compounds.

The main difference between this perspective and the one that inspired it [1] is that here, the nodes in the pathways contain either one or multiple gene-products, as stated before; whereas, in [1], each node in the retrieved pathways was only associated with one single gene-product, which is why the authors used the term protein p, instead of node N, to refer to each one of the nodes in the pathways.

Since the creation of this dataset implied data extraction from each of the pathways' nodes and edges, the *KEGGlincs* package [2, 3] was used to retrieve the KEGG Markup Language (KGML) representations of the 294 KEGG pathways obtained in the previously created *KEGG-Pertinence* dataset. These KGML representations consist of *igraph* objects [4], where each *igraph* object contains the nodes and connectivity information of each of the pathways. Therefore, an instance or ageing-related reference node N_{ref} in a KEGG pathway is a node that contains the ageing-related gene of interest, while the features for that instance are the remainingnodes in the pathway (which contains other gene-products that may be related to ageing, DR, or none of them) whose computation score is explained in the following paragraphs.

Feature values in this dataset quantify how each N_{ref} influences all the other nodes in the pathway (*i.e.*, feature nodes) [1]. The idea is that ageing-related nodes that have a common influence on a set of downstream nodes share a similar function. For each pathway, the influence score of N_{ref} on a given node N has the minimum value of 0 when the reference node N_{ref} does not influence N at all, because N is not downstream of (*i.e.*, cannot be reached from) N_{ref}. On the other hand, the score for a given node N that is downstream of N_{ref} has the maximum value of 1 if, when N_{ref} is removed from the pathway, the downstream node N becomes unreachable from the nodes that are not downstream nodes of N_{ref}. In addition, if the score of a given node N that is downstream of N_{ref} has a value

of 0.5, it means that N_{ref} accounts for half of the influence that the downstream protein is downstream of N_{ref} has a value of 0.5, it means that N_{ref} accounts for half of the influence that the downstream protein N receives. A complete description of this calculation is provided in [1], where the concept of protein p is used instead of node N.

The influence calculation is repeated for every N_{ref} and N nodes in every KEGG pathway retrieved for the KEGG-Pertinence dataset and the results were merged in such a way that, if a reference node N_{ref} has different influence score values over N for different pathways, the scores are summed, allowing for values greater than 1, which breaks the interpretation given in the last paragraph, where values lie within the interval [0,1]. In order to recover this interpretation, all the final scores are then divided by the total number of KEGG pathways used for the analysis (312). Thus, a final score for the pair $\{N_{ref}, N\}$ is the mean influence that N_{ref} exerted on N across all the pathways. The resulting dataset, which contained 1770 nodes as features and 799 ageing-related nodes as references, was called the KEGG-Influence dataset.

S.1.2 Protein descriptors

The features used for the protein descriptors dataset are as follows:

Sequence Length and Molecular Weight The sequence length is simply the count ofamino acids of a protein. The molecular weight is the sum of the molecular weights of all amino acids in the protein.

Amino Acid, Dipeptide, and Tripeptide Composition It describes the fraction of each amino acid type as well as every possible dipeptide and tripeptide combinationswithin a protein sequence, giving rise to 20,400, and 8000 features, respectively.

Z-values The z-values, also known as Sandberg descriptors [5], are the principal components of 26 different physicochemical measured and calculated properties of amino acids, and essentially represent hydrophobicity/hydrophilicity (z_1), steric/ bulk properties and polarizability (z_2), polarity (z_3), and electronic effects (z_4 and z_5) of the amino acids.

Local Descriptors Local descriptors are computed based on the variation of functional groups defined by the occurrence of specific amino acids within the primary sequence of each protein [6–8]. These functional groups are defined based on the following physicochemical properties: Hidrophobicity, Normalised Van Der Waals volume, Polarity, Polarizability, Charge, Secondary structure and Solvent Accessibility. For each one of these properties, three groups are defined according to Table S1.

There are three types of local descriptors: Composition, Transition and Distribution:

- *Composition:* Accounts for the percentage composition (relative frequency) of a particular functional group within the amino acid sequence. Therefore, there are three composition features, one for each functional group of amino acids.

- *Transition:* Transition features represent the relative frequency in which an amino acid from a particular functional group is followed by an amino acid from another functional group. More precisely, the following transitions are considered: Group1 → Group2 or Group2 → Group1; Group1 → Group3 or Group3 → Group1; and Group2 → Group3 or Group3 → Group2.
- *Distribution:* Distribution features are computed based on the percentage of how many amino acids with a particular functional group are present on the first, 25%, 50%, 75% and 100% of the amino acid sequence.

In total, local descriptors in this dataset are composed of 21 features (3 compositions, 3 transitions, 15 distributions) for each of the seven properties, which was equivalent to 147 features. When adding the Z-values (5 features), amino acid composition (20 features), sequence length, and molecular weight (2); the final dataset contained 174 features.

S.2 Hyperparameters grid

A brief description of the tuned hyperparameters of the classifiers is as follows:

- *Sampling Strategy:* It is the ratio of the number of instances associated with the minority class (*i.e.*, $Ageing_{DR}$-related genes) to the number of instances associated to the majority class (*i.e.*, $Ageing_{NotDR}$-related genes) after re-sampling. This is expressed as

$$\propto_{us} = \frac{N_m}{N_{rM}}, \quad \text{(S.1)}$$

  were N_m is the number of instances in the minority class and N_{rM} is the number of instances in the majority class after resampling.
- *Max. Features:* The number of randomly sampled features, from all *n* features in the dataset, assigned as candidate features for selection in each node of a decision tree in the forest.
- *Num. Estimators:* The number of trees in the forest.
- *Class weights:* For each class label *y* across the target labels, the *Balanced* mode computes a class weight W_y that is proportionally inverse to the number of occurrences of *y* within the training set as follows:

$$W_y = \frac{Num.Instances}{Num.Classes \times ocurrences\ of\ y}, \quad \text{(S.2)}$$

  The *Balanced_subsample* mode is the same as *Balanced* mode except that weights are computed based on the bootstrap sample for every tree grown. If this hyperparameter is set to *None*, all class labels are assigned the weight 1.
- *Binary Threshold:* It is the minimum number of occurrences that the minority value of a binary feature is required to have for that feature to be kept by a threshold-based filtering algorithm. This only applies to binary features.

Given the previous definitions, the grid in Table S2 was established. Itis to be noted that these parameters were not applicable or customisable to all classifiers. In case they do not, and for all the remaining customisable parameters provided by the ML libraries that do not appear in Table S2, default parameters were kept.

S.3 KEGG-based features importance

S.3.1 KEGG-Pertinence

Table S4 shows feature importance results for the {KEGG-Pertinence, XGB} model. The relevance gotten by Autophagy-animal, was considerably higher than the remaining relevant features, even compared to the second most relevant one (Longevity regulating pathway). On the other hand, a closer look at this feature reveals that the Autophagy pathway contains the largest number of both $Ageing_{DR}-$ and $Ageing_{NotDR}$-related genes. The *Glucagon* and the *Fluid shear stress and atherosclerosis* pathways got relevant positions despite their null significant association to either $Ageing_{DR}$-related or $Ageing_{NotDR}$-related genes, being closely followed by the AMPK pathway which neither got significant association but achieved at least an adjusted pvalue lower than 1.

S.3.2 KEGG-Influence

Table S5 shows feature importance results for the {KEGG-Influence, BRF} model. Genes are grouped in nodes that share the same values for Score, $Ageing_{DR}/Ageing_{NotDR}$ mean influence and adjusted pvalue, as described in Supplementary materials (S.1.1). The top three modules that achieved score above our defined minimum threshold of 50 didn't show any significant association. It was also interesting that none of the genes contained within the third most relevant node {PDE6A, PDE6G and PDE6B} were annotated in either GenAge nor GenDR, opening field for further research. The length of the table was extended to the first ten nodes to highlight the first three features that show significant mean influence from $Ageing_{DR}$-related genes: {PRKCA, PRKCB, PRKCG}, {PLCB1, PLCB2, PLCB3, PLCB4} and {EIF4E, EIF4E1B, EIF4E2}. From these, {PRKCA, PRKCB, PRKCG} and {EIF4E, EIF4E1B, EIF4E2} are not yet annotated in either GenAge nor GenDR. On the other hand, {PLCB1, PLCB2, PLCB3, PLCB4} is highlightable due to its extreme significant association towards $Ageing_{DR}$-related genes. From all the genes composing this last node, only PLCB4 is currently annotated in GenAge and GenDR.

S.4 Analysis of GenDR (Gene expression) genes based on DR-association probability

We contrasted our estimated DR-association probabilities against pertinence to the GenDR (Gene Expression changes) database to see if our algorithm predicts ageing-related genes that undergo expression changes during CR. The following terminology is used:

- DR_{GM}-related genes: the 152 human orthologs genes retrieved from mice, fruit fly, nematodes and yeast in GenDR (Gene Manipulations). Manipulation of these genes is associated with modifications of DR-related pro-longevity effects [9]. These genes are the "DR-related genes" reported in the main text.
- DR_{GE}-related genes: 143 human orthologs genes retrieved from the 174 mice genes with significant expression changes due to DCR in GenDR (Gene Expression). Among the 143 human genes, 78 were up-regulated and 47 down-regulated. For mice, 101 were up-regulated and 73 down-regulated [9, 10].
- DR_{GM}-probability: probability of any given gene to be DR_{GM}-related according to the arithmetic mean of the computations given by {GO terms,

BRF} and {PathDIP, CAT}. This is the same DR-probability reported in the main text.

We checked for the DR_{GE}-related genes that are also ageing-related (Figure S3) as their *DR_{GM}*-probability was already computed from the {GO terms, BRF} and {PathDIP, CAT} models. Only 18 out of 143 DR_{GE}-related genes overlapped with the ageing-related genes. The overlap between DR_{GE}-related with DR_{GM}-relatedgenes is even smaller (9 genes). We then retained only 16 genes that had entries for both GO terms and PathDIP pathways.

Results (Table S3) indicate that only 7 ageing-related genes got a DR_{GM}-probability score equal or above 0.50 and thus were classified as DR_{GM}-related. IRS2 got the highest DR_{GM}-probability across the DR_{GE}-related genes. This gene is also one of the top DR_{CM}-related gene candidates reported in the main text and the only DR_{GE}-related gene that overlapped with the top DR_{GM}-candidates, suggesting that beyond being up-regulated during DR, manipulation of this gene may also be associated with changes on the ageing-related effects associated with DR. The following three top DR_{GE}-related genes classified as DR_{GM}-related (*GCK,FMO3* and *MAT1A*) were actually DR_{GM}-related as they are already within the GenDR (Gene Manipulations) database. The remaining 3 of these 7 terms (*GYS2, INMT, ARNLT*) were just weakly DR_{GM}-predicted as they got a score equal or just above the 0.5 threshold.

References

1. Fabris, F., Freitas, A.A.: New KEGG pathway-based interpretable features for classifying ageing-related mouseproteins. Bioinformatics **32**(19), 2988–2995 (2016)
2. White, S., Medvedovic, M.: KEGGlincs Design and Application: an R Package for Exploring Relationships inBiological Pathways. (2016). Version 1; not peer reviewed. https://doi.org/10.7490/f1000research.1113436.1
3. White, S., Medvedovic, M.: Visualize All Edges Within a KEGG Pathway and Overlay LINCS data. (2016). Rpackage version 1.1.0. http://www.bioconductor.org/packages/KEGGlincs
4. Csardi, G., Nepusz, T.: The igraph software package for complex network research. InterJournal **ComplexSystems**, 1695 (2006)
5. Sandberg, M., Eriksson, L., Jonsson, J., Sjostrom, M., Wold, S.: New chemical descriptors relevant for the design of biologically active peptides. a multivariate characterization of 87 amino acids. J Med Chem **41**(14),2481–91 (1998)
6. Silla, C., Freitas, A.A.: Selecting different protein representations and classification algorithms in hierarchicalprotein function prediction. Intelligent Data Analysis **15**(6), 979–999 (2011)
7. Dubchak, I., Muchnik, I., Holbrook, S.R., Kim, S.H.: Prediction of protein folding class using global descriptionof amino acid sequence. Proceedings of the National Academy of Sciences **92**(19), 8700–8704 (1995)
8. Dubchak, I., Muchnik, I., Mayor, C., Dralyuk, I., Kim, S.: Recognition of a protein fold in the context of thescop classification. Proteins: Structure, Function and Genetics **35**, 401–407 (1999)
9. Wuttke, D., Connor, R., Vora, R., *et al.*: Dissecting the gene network of dietary restriction to identify evolutionarily conserved pathways and new functional genes, build 4 (24/06/2017). PLoS Genetics **8**(8),1002834 (2012)
10. Plank, M., Wuttke, D., van Dam, S., Clarkeab, S.A., de Magalhaes, J.P.: A meta-analysis of caloric restrictiongene expression profiles to infer common signatures and regulatory mechanisms. Mil BioSyst **9**, 1339–1349 (2012)

Supplementary Tables

Table S1

Property number	Property name	Group 1	Group 2	Group 3
Prop.1	Hydrophobicity	Polar<br>R, K, E, D, Q, N	Neutral<br>G, A, S, T, P, H, Y	Hydrophobicity<br>C, L, V, I, M, F, W
Prop.2	Normalised van der Waals Volume	0-2.78<br>G, A, S, T, P, D, C	2.95-4.0<br>N, V, E, Q, I, L	4.03-8.08<br>M, H, K, F, R, Y, W
Prop.3	Polarity	4.9-6.2<br>L, I, F, W, C, M, V, Y	8.0-9.2<br>P, A, T, G, S	10.4-13.0<br>H, Q, R, K, N, E, D
Prop.4	Polarizability	0-1.08<br>G, A, S, D, T	0.128-0.186<br>C, P, N, V, E, Q, I, L	0.219-0.409<br>K, M, H, F, R, Y, W
Prop.5	Charge	Positive<br>K, R	Neutral<br>A, N, C, Q, G, H, I, L, M, F, P, S, T, W, Y, V	Negative<br>D, E
Prop.6	Secondary Structure	Helix<br>E, A, L, M, Q, K, R, H	Strand<br>V, I, Y, C, W, F, T	Coil<br>G, N, P, S, D
Prop.7	Solvent Accessibility	Buried<br>A, L, F, C, G, I, V, W	Exposed<br>R, K, Q, E, N, D	Intermediate<br>M, S, P, T, H, Y

Table S2

Hyperparameter	Values	Algorithm
Sampling strategy	$\propto_{us} = 1$	BRF, EEC
Max features	n, $log_2(n)$	BRF, XGB
Num. estimators	500	BRF, EEC, CAT
Class weights	None, Balanced, Balanced subsample	BRF
Binary Threshold	3, 4, 5	ALL

Table S3

Gene	GenAge	GenDR Genetic Manipulations	GenDR Expression Changes		Predicted DR_{GM}-probability
			Pertinence	Regulation	
IRS2	✓	·	✓	Up	0.80
GCK	✓	✓	✓	Down	0.75
FMO3	✓	✓	✓	Up	0.64
MAT1A	✓	✓	✓	Up	0.57
GYS2	✓	·	✓	Up	0.54
INMT	✓	·	✓	Up	0.50
ARNTL	✓	·	✓	Down	0.50
GHR	✓	✓	✓	Down	0.46
PDIA3	✓	·	✓	Down	0.45
PER2	✓	·	✓	Up	0.44
CYP2F1	✓	·	✓	Down	0.37
ACLY	✓	·	✓	Down	0.36
HSPA5	✓	·	✓	Down	0.31
ALDH1A1	✓	·	✓	Up	0.31
CYP2E1	✓	·	✓	Up	0.30
DECR2	✓	·	✓	Up	0.27

Table S4.

Feature	Definition	Score	$Ageing_{DR}$ (%)	$Ageing_{NotDR}$ (%)	Ajusted pvalue
hsa04140	Autophagy - animal	100	21.28% {20/94}	5.53% {39/705}	**3.32e-05**
hsa04213	Longevity regulating pathway - multiple species	74.439	14.89% {14/94}	3.26% {23/705}	**4.36e-04**
hsa04730	Long-term depression	71.749	10.64% {10/94}	2.13% {15/705}	**8.73e-04**
hsa04152	AMPK signaling pathway	59.193	13.83% {13/94}	3.12% {22/705}	1.71e-03
hsa05146	Amoebiasis	56.054	11.7% {11/94}	1.42% {10/705}	**8.83e-06**
hsa04922	Glucagon signaling pathway	54.26	10.64% {10/94}	3.83% {27/705}	1
hsa05418	Fluid shear stress and atherosclerosis	52.466	5.32% {5/94}	1.99% {14/705}	1

Table S5

Feature	GenAge	GenDR	Score	$Ageing_{DR}$ mean influence	$Ageing_{NotDR}$ mean influence	Ajusted pvalue
PIK3C3	✓	.	100	0.05	0.01	0.047
GABARAP<br>GABARAPL2<br>GABARAPL1	✓<br>✓<br>✓	.<br>.<br>✓	59.665	0.08	0.05	0.293
PDE6A<br>PDE6G<br>PDE6B	.<br>.<br>.	.<br>.<br>.	52.638	0.02	0	0.005
CRTC2	.	.	46.685	0.03	0.02	1
RELA	.	.	42.552	0	0.04	0.087
PRKCA<br>PRKCB<br>PRKCG	.<br>.<br>.	.<br>.<br>.	37.014	0.23	0.05	**4.55e-09**
EFL1	.	.	36.089	0.01	0.04	0.241
RRAGD<br>RRAGC	✓<br>✓	.<br>.	33.622	0.02	0.01	0.425
PLCB1<br>PLCB2<br>PLCB3<br>PLCB4	.<br>.<br>.<br>✓	.<br>.<br>.<br>✓	33.456	0.81	0.05	**5.50e-86**
EIF4E<br>EIF4E1B<br>EIF4E2	.<br>.<br>.	.<br>.<br>.	33.404	0.22	0.05	**8.17E-09**

Supplementary Figures

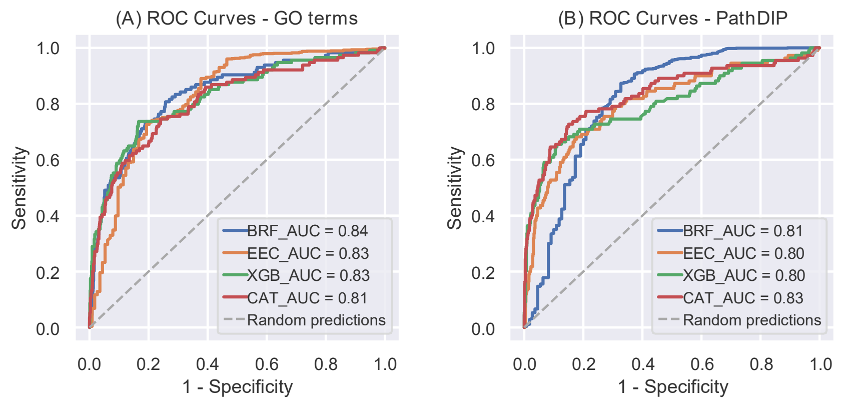

Figure S1.

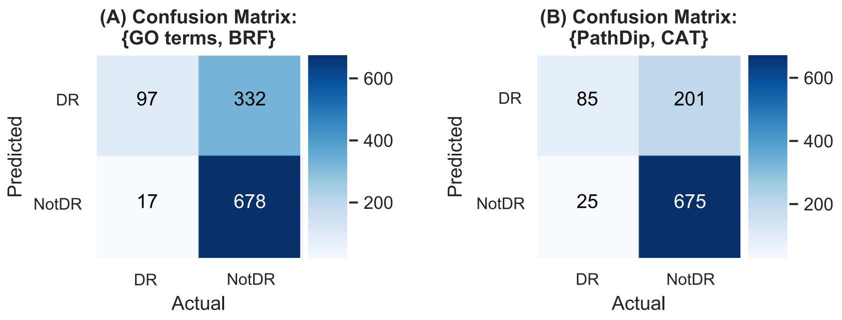

Figure S2.

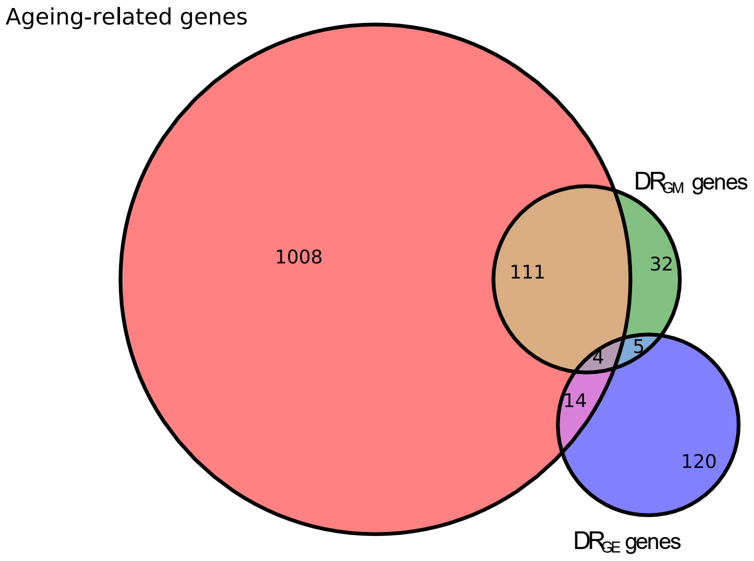
Ageing-related genes
DRGM genes
1008
111
32
4
5
14
120
DRGE genes

Figure S3.
